# Buffalo Medical and Surgical Association

**Published:** 1885-09

**Authors:** 


### Society Reports.

### Buffalo Medical and Surgical Association.

*Meeting of July 7th, 1885.*

The President, Dr. Charles G. Stockton, in the chair; Dr. Frank H. Potter, Secretary.

The minutes of the previous meeting were read and approved, after a correction by Dr. Bartlett. He did not wish to be understood as condemning irrigation in pelvic abscesses, but he wished to say that he had seen good results when the abscess cavity was not washed out by antiseptic solutions.

Dr. Roswell Park read a paper, entitled, "The Treatment of Gunshot Wounds."

Dr. Heath opened the discussion by endorsing the principles of treatment as advocated by the essayist. He said the general practitioner did not fully appreciate the importance of leaving these wounds alone. Probing wounds as a rule was injurious, but sometimes, and in some situations, it was proper treatment, as, for instance, in some injuries to the hand.

Dr. Cronyn also endorsed the views of the essayist. It was only in exceptional cases that probing was necessary or justifiable. He then related several cases which, by the use of the probe, he had been enabled to treat successfully, when without it he thought a favorable result would have been very doubtful. It was a generally accepted principle that probing was harmful.

Dr. Tremaine had some very pronounced views on this subject. As he was about to publish these in the forthcoming "Handbook of Medicine," of Wm. Wood & Co., he would only say, at this time, that in the main he agreed with the essayist. He requested all physicians to keep all probes and fingers from the wounds of patients who were to be sent to the hospitals with which he was connected.

Dr. Wetmore said when the ball was jagged it should be removed, if possible. When probing, fingers and instruments should always be disinfected. He believes that the police should be instructed in the primary dressing of wounds; that if this were done, many cases might be saved. He called especial attention to injury to nerves, and that such injuries required the probing of the wound.

Dr. Coakley was not prepared to agree with the essayist. The methods of treatment advocated were a departure from those recommended by most of the authors, and established by his experience. Serious results might follow from this let-alone practice. He also called attention to the danger that might follow from the free use of iodoform and carbolic acid. Many cases of poisoning had been reported.

Dr. F. W. Bartlett said that there were different methods of treatment in vogue at different times. Formerly, continued irrigation was the practice, now it is the dry method; for himself, he favors the latter.

Dr. W. W. Potter said that, speaking as an army surgeon during the late war, he wished it known that there was a larger percentage of wounded saved then than during any previous time. He believed he was one of the earliest surgeons to discountenance the use of the probe, and was prepared to say that it would have been better for the soldiers if there had been no probes in the army. In the service, transportation was often a potent factor contributing to a fatal result. He advocated the use of iodoform, and thinks its judicious use would never be followed by poisoning. The medical man first seeing a wound, should let it alone until he was able to do all that was required. Bullets in motion are dangerous, and, at rest, harmless. Sometimes it was necessary to remove bullets, and cited cases of injury to the chest and abdomen in which this had been the proper treatment.

The essayist said that the last editions of the prominent works on surgery all advocated the principles of treatment under consideration. It was possible for iodoform to cause poisoning, but it had been used in enormous quantities with impunity ; on the other hand, a small quantity sometimes caused unfavorable symptoms.

Dr. Bartlett asked who was the first surgeon to advocate the principles of the occlusion of wounds.

Dr. Potter said that Dr. Howard, of the regular army, was among its earliest advocates.

Dr. Cronyn thought it was Gutherie who first practiced that method in the Peninsular war.

Dr. Tremaine said the credit of this method must be given to Sir Sinclair Digby, as he ante-dated all the others in the use of occlusion in wound-treatment.

Dr. Park then reported a case of total extirpation of the larynx, for epithelioma, in a patient aged 64. He gave a detailed account of the operation which occurred two weeks previous, and reported the patient doing well. The case occurred in the practice of Dr. Hinkle, who early recognized the condition, and advised removal.

Great interest attaches to the case, as it was the third complete extirpation performed in this country—the other one had lived several months ; the second dying on the fourth day.

Dr. Hopkins wished to call the attention of the Association to the first annual report of the Hospital of the Sisters of Charity and Branch Emergency Hospital, as in that report he found statements which, in his opinion, merited investigation.

Dr. Cronyn protested vigorously against the Association considering the matter in any way whatever. It was a question entirely beyond its province.

After some further discussion as to the jurisdiction of the Association, Dr. Van Peyma moved the following committee be appointed, and that this question be referred to it, with instructions to report at the next meeting: Drs. Hopkins, Stockton, W. W. Potter, Davidson and Cary.

Dr. Crego moved that the whole question be laid upon the table, which was lost.

The Chair then stated that he desired the Association to manifest its pleasure in this matter by motion, otherwise he should feel it his duty to declare the question out of order.

No motion to this effect being made, he so declared. Whereupon Dr. Hopkins saying, that, as a majority of the Association did not seem to be in accord with the decision of the Chair, he would appeal from that decision.

The appeal being seconded, the question was put to the house by the Vice-President, who first ruled that it would require a two-thirds vote to sustain the appeal, and that all members present must vote.

The question being put, the vote stood twenty-three (23) for the appeal and twelve (12) against. The majority failing to obtain the requisite two-thirds, the appeal was declared lost and the Chair sustained.

Dr. Cary then revived Dr. Van Peyma's motion for the appointment of a committee of investigation, consisting of the gentlemen mentioned, which was carried.

The Association then adjourned.

